# Bidirectional association between asthma and otitis media in children

**DOI:** 10.1186/s13223-020-00500-7

**Published:** 2021-01-09

**Authors:** So Young Kim, Hye-Rim Kim, Chanyang Min, Hyo Geun Choi

**Affiliations:** 1 Department of Otorhinolaryngology-Head & Neck Surgery, CHA Bundang Medical Center, CHA University, Seongnam, Korea; 2 Department of Pediatrics, CHA Bundang Medical Center, CHA University, Seongnam, Korea; 3 Hallym Data Science Laboratory, Hallym University College of Medicine, Anyang, Korea; 4 Graduate School of Public Health, Seoul National University, Seoul, Korea; 5 Department of Otorhinolaryngology-Head & Neck Surgery, Hallym University Sacred Heart Hospital, 22, Gwanpyeong-ro 170beon-gil, Dongan-guGyeonggi-do, Anyang, 14068 Republic of Korea

**Keywords:** Asthma, Otitis media, Cohort studies, Epidemiology

## Abstract

**Background:**

This study explored the reciprocal association between otitis media and asthma in children.

**Methods:**

The 2002–2013 Korean Health Insurance Review and Assessment Service-National Sample Cohort participants < 15 years old were used. In study I, 14,665 asthma patients from 2002 through 2005 were selected. The asthma patients were matched 1:1 with the control I group, and the occurrence of otitis media was followed until 2013. In study II, 27,043 otitis media patients from 2002 through 2005 were selected. The otitis media patients were matched 1:1 with the control II group, and the occurrence of asthma was followed until 2013. Stratified Cox proportional hazard models were used to analyze the hazard ratio (HRs) of asthma for otitis media (study I) and otitis media for asthma (study II).

**Results:**

The HR for otitis media was 1.46 in asthma patients (95% confidence interval [CI] = 1.40–1.52, P < 0.001). The HR for asthma was 1.43 in otitis media patients (95% confidence interval [CI] = 1.36–1.50, P < 0.001).

**Conclusion:**

Asthma and otitis media have a bidirectional association in children.

## Background

Otitis media refers to middle ear inflammation due to both viral and bacterial infections. In addition to acute or chronic otitis media, otitis media can be accompanied by effusion, which is caused by the dysfunction of the Eustachian tube. Because of the anatomical features of developing Eustachian tubes in children, many children suffer from recurrent otitis media. It was reported that the prevalence of otitis media with effusion was approximately 50% in children < 1 year old and 60% in children 2 years old [1]. Persistent or recurrent otitis media in children could result in hearing loss and chronic middle ear inflammatory conditions. Thus, prompt management and prevention of the recurrence of otitis media are crucial in children. In addition to viral or bacterial infection and Eustachian tube dysfunction, several prior studies suggested the relationship of otitis media with allergies [2, 3]. One specific type of otitis media, eosinophilic otitis media, is caused by eosinophilic inflammation and is related to asthma [4].

Asthma is the most common lower airway disease in children [5]. Approximately 8.3 – 9.3% of children 0 – 17 years old have asthma in the US [5]. In Korea, approximately 6.27 – 7.39% of 4 – 12-year-old children have asthma [6]. Asthma patients often also have upper airway inflammatory disorders, such as rhinitis [7, 8]. Based on the continuity of the respiratory mucosa and inflammatory response, united airway disease was postulated to explain the co-occurrence of airway diseases [9]. Because of the connection of the middle ear to the airway via the Eustachian tube, the occurrence or recurrence of otitis media might increase the risk of asthma. Especially in children, the immaturity of the Eustachian tube could be more involved in the potential for the development of asthma in children with otitis media. In line with this, previous studies reported a high risk of asthma in children with otitis media [10, 11].

This study hypothesized that the risk of otitis media might be high in asthmatic children and that otitis media could be a predictive factor for asthma. To evaluate this hypothesis, asthmatic children and children with otitis media were followed for the occurrence of otitis media and asthma, respectively. To avoid confounders for the risk of otitis media, the participants with susceptibility to otitis media due to anatomical causes, for instance facial anomaly and cleft lip and palate, were excluded from this study. In addition, control groups were matched with study groups for both demographic and socioeconomic factors, because the risk of otitis media could be influenced by demographic factors, such as age and environmental factors, such as air pollutants [12]. Other allergic diseases, namely, atopic dermatitis and upper airway inflammatory diseases such as sinusitis, were used as adjustment factors to attenuate confounding effects. This is the first study to investigate the reciprocal association between asthma and otitis media.

## Methods

### Study population and data collection

This national cohort study relied on data from the Korean Health Insurance Review and Assessment Service-National Sample Cohort (HIRA-NSC). The detailed description of this data was described in our previous studies (Additional file 1: S1 Description) [13, 14].

### Study I

Of 1,125,691 patients with 104,168,614 medical claim codes from 2002 through 2013, we excluded patients with any cancer histories (International Classification of Diseases [ICD]-10 codes of C00-D48, n = 183,836), facial anomalies (Q10-Q18, n = 2487), and cleft lips and palates (Q35-Q37, n = 245). Among all asthmatic patients (n = 190,895), we only included asthma diagnosed from 2002 through 2005 to enable a long follow-up period. Therefore, we excluded asthma patients who were newly diagnosed from 2006 through 2013 (n = 100,293). The participants with a history of otitis media before the index date were excluded from the asthma group (n = 16,124). Among asthma patients, we excluded participants ≥ 15 years old (n = 35,603) and 0–4 years old (n = 24,210).

**Fig. 1:**
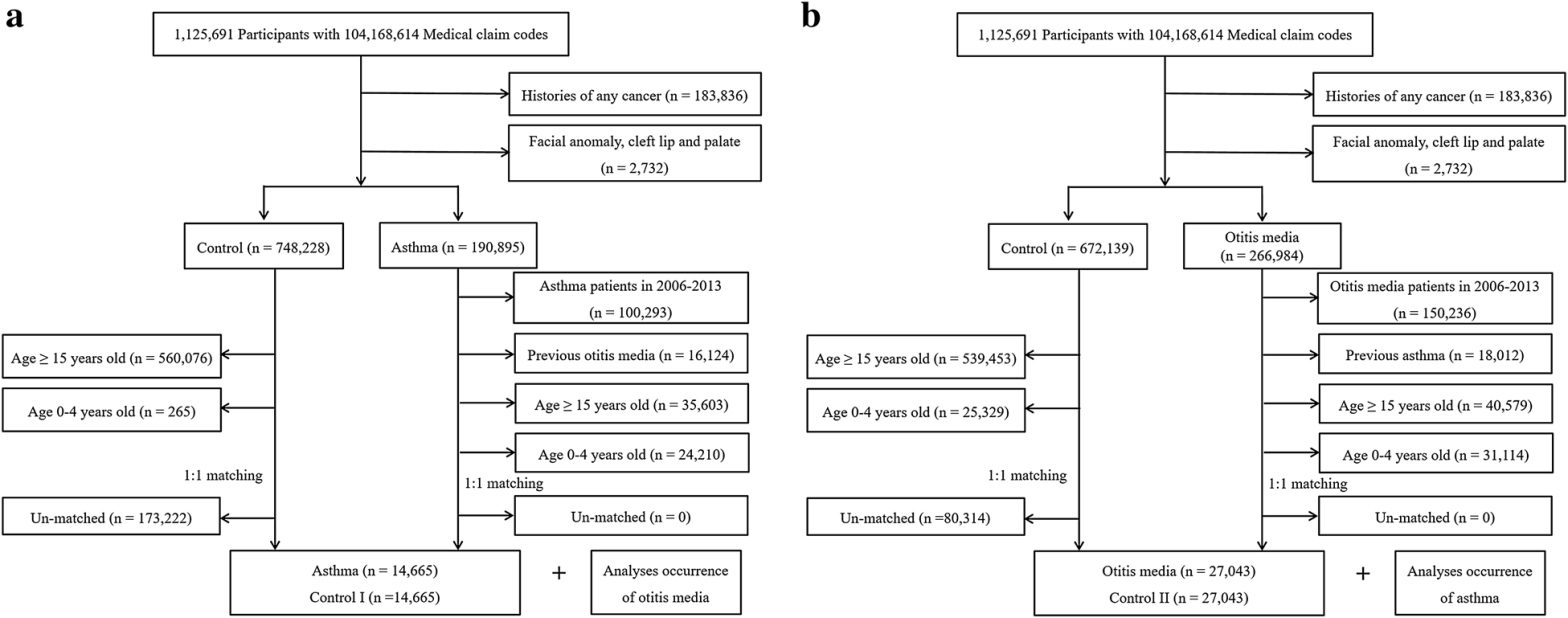
A schematic illustration of the participant selection process used in the present study. **a** Of a total of 1,125,691 participants, 14,665 asthma patients were matched with 14,665 control I participants for age, group, sex, income group, and region of residence. **b** Of a total of 1,125,691 participants, 27,043 otitis media patients were matched with 27,043 control II participants for age, group, sex, income group, and region of residence

The asthma group was matched 1:1 with participants (control I group) who were not diagnosed with asthma from 2002 through 2013. The control I group was selected from the total population (n = 748,228). Among them, we excluded participants ≥ 15 years old (n = 560,076) and 0–4 years old (n = 265). Matching was performed for age group, sex, income group, and region of residence. To prevent selection bias when selecting the matched participants, the control participants were sorted using a random number order and were then selected from top to bottom. We set the index date as the date of the diagnosis of asthma. It was assumed that the matched control participants were involved at the same time as the asthma participants (index date). Therefore, the control participants who died before the index date or who had histories of otitis media before the index date were excluded. Both the asthma and control I cohorts were followed to December 31, 2013, or the date of death. Finally, 1:1 matching resulted in the inclusion of 14,665 asthma patients and 14,665 control participants (Fig. 1a).

The mean follow-up time was 88.6 months (standard deviation [SD] = 52.2) in the asthma group and 100.0 months (SD = 48.3) in the control I group.

### Study II

Of 1,125,691 patients with 104,168,614 medical claim codes from 2002 through 2013, we excluded patients with any cancer histories (n = 183,836), facial anomalies (n = 2487), and cleft lips and palates (n = 245), as in study I. Among all otitis media patients (n = 266,984), we excluded otitis media patients newly diagnosed from 2006 to 2013 year for the same reason as in study I (n = 150,236). The participants with a history of asthma before the index date were excluded from the otitis media group (n = 18,012). Among otitis media patients, we excluded participants ≥ 15 years old (n = 40,579) and 0–4 years old (n = 31,114).

The otitis media group was matched 1:1 with participants (control II group) who were not diagnosed with otitis media from 2002 through 2013. The control II group was selected from the total population (n = 672,139). Among them, we excluded participants ≥ 15 years old (n = 539,453) and 0–4 years old (n = 25,329). Matching was performed for age group, sex, income group, and region of residence. To prevent selection bias when selecting the matched participants, the control participants were sorted using another random number order and were then selected from top to bottom. We set the index date as the date of the diagnosis of otitis media. It was assumed that the matched control participants were involved at the same time as the otitis media participants (index date). Therefore, the control participants who died before the index date or who had histories of otitis media before the index date were substituted. Both otitis media and control II participants were followed to December 31, 2013, or the date of death. Finally, 1:1 matching resulted in the inclusion of 27,043 otitis media patients and 27,043 control participants (Fig. 1b).

The mean follow-up time was 109.5 months (SD = 39.9) in the otitis media group and 114.5 months (SD = 35.0) in the control II group. Additionally, we analyzed the occurrence of asthma in patients with otitis media histories ≥ 2 times and ≥ 3 times during from 2002 through 2005 and in the matched controls.

### Variables

Asthma participants were selected as those with a diagnosis of asthma (ICD-10: J45) or status asthmaticus (J46). Among them, we selected participants who were diagnosed with asthma by a physician more than 2 times and who were treated with asthma-related medications, including inhaled corticosteroids (ICSs), ICSs combined with long-acting β2-agonists (LABAs), oral leukotriene antagonists (LTRAs), short-acting β2-agonists (SABAs), systemic LABAs, xanthine derivatives, and systemic corticosteroids. This method has been modified from a previous study [15].

Otitis media was defined as a diagnosis by physicians using ICD-10 codes of H65-H67 at least once.

The age, sex, income, and region of residence were defined as in previous studies [16, 17] (Additional file 1: S1 Description). Atopic dermatitis (L20) was defined as being present if a participant was treated ≥ 2 times, as in a previous study [18]. Chronic rhinosinusitis (CRS) was diagnosed using ICD-10 codes (J32). Among patients with CRS, we selected the participants who were treated ≥ 2 times and those who underwent head and neck computerized tomography evaluations (Claim codes: HA401-HA416, HA441-HA443, HA451-HA453, HA461-HA463, or HA471-HA473), as in our previous study [19].

### Statistical analyses

Chi-square tests were used to compare the general characteristics between the asthma and control I groups (study I) and between the otitis media and control II groups (study II).

Stratified Cox proportional hazard models were used to assess hazard ratios (HRs) for asthma (independent variable) with respect to otitis media (dependent variable) in study I and for otitis media (independent variable) with respect to asthma (dependent variable) in study II. Crude (simple) and adjusted (for atopic dermatitis and chronic rhinosinusitis [CRS]) models were used, and 95% CIs were calculated. In these analyses, age, sex, income, and region of residence were used to stratify the populations. Kaplan–Meier analysis and the log-rank test were analyzed.

For the subgroup analyses, we divided the participants by age (0–4, 5–9, 10–14 years old) and sex (male and female) to confirm that these relations were reliable across age and sex categories. In addition, we analyzed HRs according to the frequency of otitis media as ≥ 2 times, ≥ 3 times, ≥ 4 times, and ≥ 5 times in study I, as the frequency of otitis media could be a surrogate marker of the severity of disease. Likewise, we analyzed the HRs of otitis media for asthma according to the frequency of otitis media as ≥ 2 times and ≥ 3 times in study II.

Two-tailed analyses were conducted, and P values less than 0.05 were considered to indicate significance. The results were statistically analyzed with SPSS version 22.0 (IBM, Armonk, NY, USA) and SAS version 9.4 (SAS Institute Inc., Cary, NC, USA).

## Results

### Study I

The duration from index date to development of otitis media (≥ 1 time) was 34.1 months (SD = 33.4) in the asthma group and 35.4 months (SD = 35.9) in the control I group. The rate of having otitis media ≥ 1 time was higher in the asthma group (41.9% [6138/14,665], P < 0.001, Table 1). In addition, the rates of having otitis media ≥ 2 times, ≥ 3 times, ≥ 4 times, and ≥ 5 times were also higher in the asthma group (each P < 0.001). The general characteristics (age, sex, income, and region of residence) of the participants were the same due to the matching procedure (P = 1.000, Additional file 2: Table S2). The rates of atopic dermatitis and CRS were higher in the asthma group than in the control I group (each P < 0.001).

**Table 1:** General characteristics of participants

Characteristics	Study I			Study II		
	Asthma (n, %)	Control I (n, %)	P-value	Otitis media (n, %)	Control II (n, %)	P-value
Atopic dermatitis	3220 (22.0)	1877 (12.8)	< 0.001*	4753 (17.6)	3306 (12.2)	< 0.001*
Chronic sinusitis	758 (5.2)	452 (3.1)	< 0.001*	1373 (5.1)	646 (2.4)	< 0.001*
Otitis media ≥ 1 time	6138 (41.9)	4418 (30.1)	< 0.001*	27,043 (100.0)	0 (0.0)	< 0.001*
Otitis media ≥ 2times	4009 (27.3)	2652 (18.1)	< 0.001*	18,723 (69.2)	0 (0.0)	< 0.001*
Otitis media ≥ 3 times	2765 (18.9)	1751 (11.9)	< 0.001*	13,317 (49.2)	0 (0.0)	< 0.001*
Otitis media ≥ 4 times	2062 (14.1)	1244 (8.5)	< 0.001*	10,063 (37.2)	0 (0.0)	< 0.001*
Otitis media ≥ 5 times	1565 (10.7)	929 (6.3)	< 0.001*	7805 (28.9)	0 (0.0)	< 0.001*
Asthma	0 (0.0)	14,665 (100.0)	< 0.001*	4468 (16.5)	3086 (11.4)	< 0.001*

* Chi-square test. Significance at P < 0.05

**Fig. 2:**
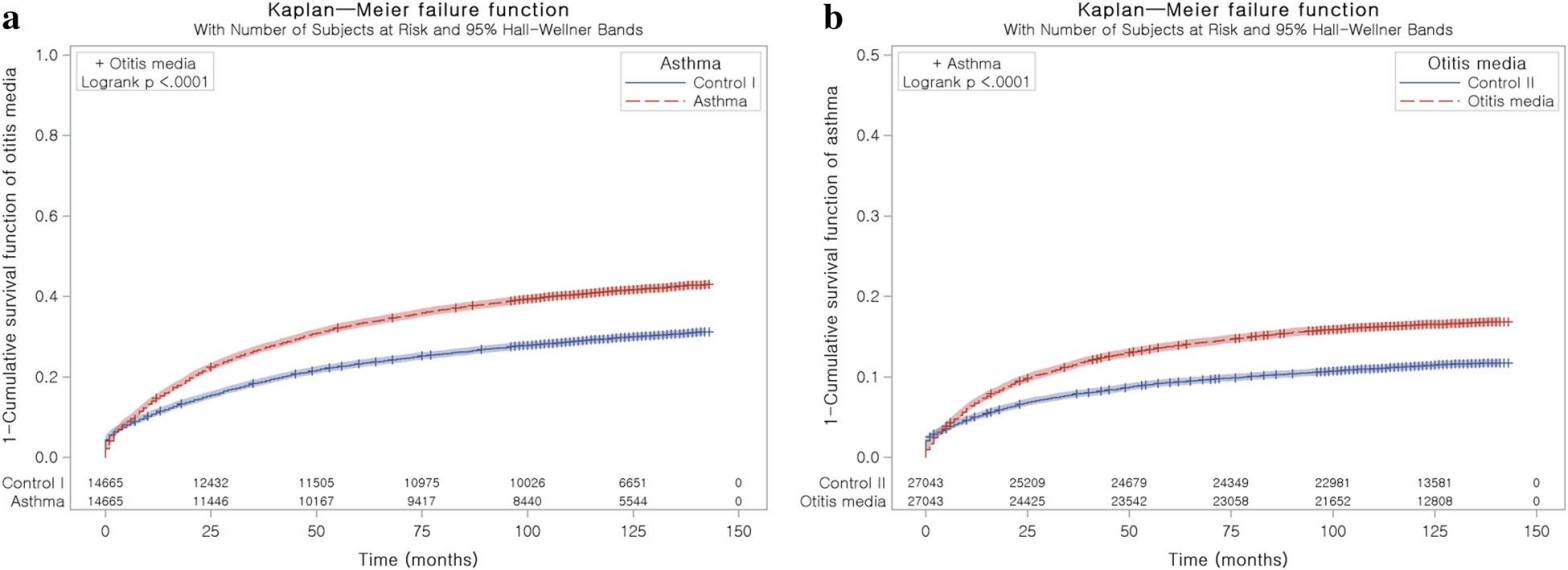
**a** Kaplan Meier curve of asthma for otitis media. It was explained as 1—survival function curve. **b** Kaplan Meier curve of otitis media for asthma. It was explained as 1—survival function curve

The adjusted HR for having otitis media ≥ 1 time was 1.46 (95% CI = 1.40–1.52) in the asthma group compared to the control I group (P < 0.001, Table 2). Kaplan–Meier analysis showed consistent results (Fig. 2a). In subgroup analyses performed according to age and sex, all the crude and adjusted HRs for otitis media were higher in the asthma group than in the control I group (each P < 0.001). The adjusted HRs were 1.47 (95% CI = 1.40–1.53) in the group 5–9 years old, 1.42 (95% CI = 1.29–1.56) in the group 10–14 years old, 1.40 (95% CI = 1.33–1.48) in males, and 1.53 (95% CI = 1.45–1.62) in females.

**Table 2:** Crude and adjusted hazard ratios (95% confidence interval) of asthma for otitis media (≥ 1 time) according to age and sex

Characteristics	HRs for otitis media			
	Crude^a^	P-value	Adjusted^a,b^	P-value
Total participants (n = 29,330)				
Asthma	1.51 (1.45–1.57)	< 0.001*	1.46 (1.40–1.52)	< 0.001*
Control I	1.00		1.00	
Age 5–9 years old (n = 21,674)				
Asthma	1.50 (1.45–1.57)	< 0.001*	1.47 (1.40–1.53)	< 0.001*
Control I	1.00		1.00	
Age 10–14 years old (n = 7,656)				
Asthma	1.49 (1.35–1.63)	< 0.001*	1.42 (1.29–1.56)	< 0.001*
Control I	1.00		1.00	
Males (n = 16,232)				
Asthma	1.44 (1.37–1.52)	< 0.001*	1.40 (1.33–1.48)	< 0.001*
Control I	1.00		1.00	
Females (n = 13,098)				
Asthma	1.58 (1.50–1.68)	< 0.001*	1.53 (1.45–1.62)	< 0.001*
Control I	1.00		1.00	

*Cox-proportional hazard regression model, Significance at P < 0.05

^a^Stratified model for age, income, and region of residence

^b^Adjusted model for atopic dermatitis and chronic sinusitis

According to the frequency of otitis media, the findings were consistent (each P < 0.001, Table 3). The adjusted HRs were 1.60 (95% CI = 1.52–1.68) for asthma with respect to having otitis media ≥ 2 times, 1.57 (95% CI = 1.48–1.67) for asthma with respect to having otitis media ≥ 3 times, 1.63 (95% CI = 1.52–1.75) for asthma with respect to having otitis media ≥ 4 times, and 1.63 (95% CI = 1.50–1.77) for asthma with respect to having otitis media ≥ 5 times.

**Table 3:** Crude and adjusted hazard ratios (95% confidence interval) of asthma for otitis media according to frequency of otitis media

Characteristics	HRs for otitis media			
	Crude^a^	P-value	Adjusted^ab^	P-value
Otitis media ≥ 2times				
Asthma	1.60 (1.52–1.68)	< 0.001*	1.60 (1.52–1.68)	< 0.001*
Control I	1.00		1.00	
Otitis media ≥ 3 times				
Asthma	1.64 (1.54–1.74)	< 0.001*	1.57 (1.48–1.67)	< 0.001*
Control I	1.00		1.00	
Otitis media ≥ 4 times				
Asthma	1.71 (1.59–1.83)	< 0.001*	1.63 (1.52–1.75)	< 0.001*
Control I	1.00		1.00	
Otitis media ≥ 5 times				
Asthma	1.72 (1.58–1.86)	< 0.001*	1.63 (1.50–1.77)	< 0.001*
Control I	1.00		1.00	

* Cox-proportional hazard regression model, Significance at P < 0.05

^a^Stratified model for age, income, and region of residence

^b^Adjusted model for atopic dermatitis and chronic sinusitis

### Study II

The duration from the index date to the development of asthma was 29.5 months (SD = 30.6) in the otitis media group and 30.2 months (SD = 34.0) in the control II group. The rate of asthma was higher in the otitis media group (16.5% [4468/27,043]) than in the control II group (11.4% [3086/27,043], P < 0.001, Table 1). The general characteristics (age, sex, income, and region of residence) of the participants were the same due to the matching procedure (P = 1.000, Additional file 2: Table S2). The rates of atopic dermatitis and CRS were higher in the otitis media group than in the control II group (each P < 0.001).

The adjusted HR for asthma was 1.43 (95% CI = 1.36–1.50) in the otitis media group compared to the control II group (P < 0.001, Table 4). Kaplan–Meier analysis showed consistent results (Fig. 2b). In subgroup analyses performed according to age and sex, all the crude and adjusted HRs for asthma were higher in the otitis media group than in the control II group (each P < 0.001). The adjusted HRs were 1.45 (95% CI = 1.38–1.53) in the group 5–9 years old, 1.34 (95% CI = 1.20–1.49) in the group 10–14 years old, 1.42 (95% CI = 1.33–1.51) in males, and 1.44 (95% CI = 1.35–1.55) in females.

**Table 4:** Crude and adjusted hazard ratios (95% confidence interval) of otitis media (≥ 1 time) for asthma according to age and sex

Characteristics	HRs for asthma			
	Crude^a^	P-value	Adjusted^ab^	P-value
Total participants (n = 540,86)				
Otitis media	1.49 (1.42–1.56)	< 0.001*	1.43 (1.36–1.50)	< 0.001*
Control II	1.00		1.00	
Age 5–9 years old (n = 36,048)				
Otitis media	1.51 (1.43–1.59)	< 0.001*	1.45 (1.38–1.53)	< 0.001*
Control II	1.00		1.00	
Age 10–14 years old (n = 18,038)				
Otitis media	1.41 (1.27–1.56)	< 0.001*	1.34 (1.20–1.49)	< 0.001*
Control II	1.00		1.00	
Males (n = 28,582)				
Otitis media	1.47 (1.39–1.57)	< 0.001*	1.42 (1.33–1.51)	< 0.001*
Control II	1.00		1.00	
Females (n = 25,504)				
Otitis media	1.50 (1.40–1.61)	< 0.001*	1.44 (1.35–1.55)	< 0.001*
Control II	1.00		1.00	

*Cox-proportional hazard regression model, Significance at P < 0.05

^a^Stratified model for age, sex, income, and region of residence

^b^Adjusted model for atopic dermatitis and chronic sinusitis

According to the frequency of otitis media, the findings were consistent (each P < 0.001, Table 5). The adjusted HRs were 1.60 (95% CI = 1.51–1.68) for having otitis media ≥ 2 times with respect to asthma and 1.64 (95% CI = 1.54–1.74) for having otitis media ≥ 3 times with respect to asthma.

**Table 5:** Crude and adjusted hazard ratios (95% confidence interval) of otitis media for asthma according to frequency of otitis media

Characteristics	HRs for asthma			
	Crude^a^	P-value	Adjusted^a^^b^	P-value
Otitis media ≥ 2times	1.66 (1.57–1.75)	< 0.001*	1.60 (1.51–1.68)	< 0.001*
Control II	1.00		1.00	
Otitis media ≥ 3 times	1.71 (1.61–1.82)	< 0.001*	1.64 (1.54–1.74)	< 0.001*
Control II	1.00		1.00	

^*^ Cox-proportional hazard regression model, Significance at P < 0.05

^a^ Stratified model for age, sex, income, and region of residence

^b^ Adjusted model for atopic dermatitis and chronic sinusitis

## Discussion

The risk of otitis media was increased in the asthmatic children compared with the control population. In addition, the risk of asthma was elevated in the children with otitis media. These results were consistent across age and sex subgroups. In addition, the risk of recurrent otitis media was also higher in asthmatic children and vice versa.

Similar to the present results, previous studies proposed an association between otitis media and asthma [10]. In the National Health and Nutrition Examination Survey in the United States, the history of otitis media was associated with asthma in children 2–11 years old (odds ratio [OR] = 1.70, 95% CI = 1.22–2.37) [20]. However, the temporal relationship between otitis media and asthma could not be delineated due to the cross-sectional study design in that study. On the other hand, a prior study reported the inverse causality [11]. Otitis media in infancy was related to childhood asthma (OR = 1.8, 95% CI = 1.2–2.6) [11]. The present study improved on the previous findings by using two longitudinal follow-up studies. Multiple pathophysiologies might be involved in the relationship between asthma and otitis media in children.

In asthmatic children, the inflammation and mucosal swelling of the airway, including the Eustachian tube, could induce the narrowing or obstruction of the Eustachian tube, which increases the susceptibility to otitis media. Asthma patients have inflammation of the lower airway due to both T helper type 1 and T helper type 2 (Th2) immune responses according to the endotypes. This inflammation of the airway may induce the inflammation of the mucosa of the orifice of the Eustachian tube, thereby impeding the ventilation of the middle ear. Moreover, recurrent wheezing and airway secretions in asthma patients could physically obstruct the Eustachian tube. The narrowing of the Eustachian tube opening hinders the mucociliary clearance [21] and promotes accumulation of infection sources. In addition, airway inflammatory and allergic conditions could lead to susceptibility to viral or bacterial infection, resulting in otitis media. A skewed Th2 immune response and eosinophilia could elevate the risk of eosinophilic otitis media in asthmatic children. Eosinophilic otitis media has middle ear effusion rich in eosinophils, and it is prevalent among asthma patients [22]. Although the proportion of patients with eosinophilic otitis media may not be high in the pediatric population, the Th2 immune response and eosinophilia extending to the middle ear might be one of the links between otitis media and asthma.

On the other hand, middle ear inflammation in otitis media children could be a predictor of the risk of asthma due to the united airway. Several prior studies reported an increased risk of asthma following airway respiratory infection in early childhood [23–25]. A prospective birth cohort study demonstrated as high as a 7.20-fold higher number of acute airway infections in asthmatic children (95% CI = 2.49–20.88) [23]. Frequent airway infections in childhood could impair the development of airway immune function and alter the airway microbiome, thereby increasing the risk of airway hyperreactivity and asthma [26, 27].

In this study, all age and sex subgroups showed a bidirectional association between otitis media and asthma. Previous studies reported the association between otitis media and allergy in older children [28, 29]. Age was suggested as a modulating factor for the relationship between allergy and otitis media due to the development of immune and allergy with age [30]. The inclusion of both serous and mucoid otitis media in this study may contribute to the finding of an association between otitis media and asthma in all age subgroups in this study. In addition, other inflammatory and immunologic contributions, in addition to allergy, in asthmatic children might potentiate the relationship between otitis media and asthma in all age subgroups.

The asthmatic children had a higher risk of recurrent otitis media, and the children with recurrent otitis media had a higher risk of asthma in this study. This implies that vulnerability to otitis media other than that due to opportunistic viral or bacterial infection might contribute to the reciprocal relationship between otitis media and asthma in children. In fact, a previous study reported a higher prevalence of serous otitis media than mucous otitis media in asthmatic children [31]. Serous otitis media is associated with poor middle ear ventilation, while mucous otitis media is often accompanied by bacterial infections that require antibacterial medications [32]. Thus, Eustachian tube dysfunction and airway inflammation might impact the relationship between repeated otitis media and asthma in children.

This study used a large, nationwide population cohort, which increased the statistical power. The large number of cohort participants permitted the random selection of control participants who matched the study participants with regard to age, sex, income, and region of residence. Other allergic diseases, namely, atopic dermatitis and paranasal sinus inflammation due to chronic sinusitis, were used to adjust the model to eliminate their confounding effects. However, due to the retrospective study design, the causality between asthma and otitis media could not be concluded. Because this study was based on data from clinical visits or health claim codes, the potential misdiagnosis could not be totally excluded. Subclinical or spontaneously recovered otitis media might have been missed in this study In addition, the severity and management of otitis media and asthma were heterogeneous among the study population. However, to explore the impact of asthma on the severity of otitis media and the impact of the severity of otitis media on asthma, subgroup analyses were performed according to the frequency of the occurrence of otitis media. Although we matched and adjusted for potential confounders, there were still unconsidered possible confounders, such as the lifestyle factors of breast feeding and body mass index.

## Conclusion

Asthma elevated the risk of otitis media, and otitis media could be a predictive factor for asthma in children.

## Supplementary information

**Additional file 1. File S1.** Description: study population and data collection.

**Additional file 2. Table S2.** General characteristics of participants.

## Data Availability

Releasing of the data by the researcher is not allowed legally. All of data are available from the database of National health Insurance Sharing Service (NHISS)https://nhiss.nhis.or.kr/ NHISS allows all of this data for the any researcher who promises to follow the research ethics with some cost. If you want to access the data of this article, you could download it from the website after promising to follow the research ethics.

## Abbreviations

HR: Hazard ratio
CI: Confidence interval
SD: Standard deviation
ICSs: Inhaled corticosteroids
LABAs: Long-acting β2-agonists
LTRAs: Leukotriene antagonists
SABAs: Short-acting β2-agonists
CRS: Chronic rhinosinusitis
OR: Odds ratio

## References

[1] Simon F, Haggard M, Rosenfeld RM, Jia H, Peer S, Calmels MN, Couloigner V, Teissier N (2018). International consensus (ICON) on management of otitis media with effusion in children. Eur Ann Otorhinolaryngol Head Neck Dis.

[2] Hurst DS (2011). The role of allergy in otitis media with effusion. Otolaryngol Clin North Am.

[3] Chantzi FM, Kafetzis DA, Bairamis T, Avramidou C, Paleologou N, Grimani I, Apostolopoulos N, Papadopoulos NG (2006). IgE sensitization, respiratory allergy symptoms, and heritability independently increase the risk of otitis media with effusion. Allergy.

[4] Tanaka Y, Nonaka M, Yamamura Y, Tagaya E, Pawankar R, Yoshihara T (2013). Improvement of eosinophilic otitis media by optimized asthma treatment. Allergy Asthma Immunol Res.

[5] Akinbami LJ, Simon AE, Rossen LM (2016). Changing trends in asthma prevalence among children. Pediatrics.

[6] Jang Y, Shin A (2015). Sex-based differences in asthma among preschool and school-aged children in Korea. PLoS ONE.

[7] de Oliveira TB, Moscon JG, Ferreira E, da Veiga ABG (2019). Prevalence of symptoms of asthma and allergic rhinitis in children in Southern Brazil: a ten-year monitoring study. J Asthma.

[8] Papadopoulou A, Tsoukala D, Tsoumakas K (2014). Rhinitis and asthma in children: comorbitity or united airway disease?. Curr Pediatr Rev.

[9] Bellanti JA, Settipane RA (2014). United airway disease. Allergy Asthma Proc.

[10] MacIntyre EA, Heinrich J (2012). Otitis media in infancy and the development of asthma and atopic disease. Curr Allergy Asthma Rep.

[11] Nafstad P, Magnus P, Jaakkola JJ (2000). Early respiratory infections and childhood asthma. Pediatrics.

[12] Park M, Lee JS, Park MK (2019). The effects of air pollutants on the prevalence of common ear, nose, and throat diseases in South Korea: a national population-based study. Clin Exp Otorhinolaryngol.

[13] Kim SY, Kim HJ, Lim H, Kong IG, Kim M, Choi HG (2018). Bidirectional association between gastroesophageal reflux disease and depression: Two different nested case-control studies using a national sample cohort. Sci Rep.

[14] Kim SY, Lim JS, Kong IG, Choi HG (2018). Hearing impairment and the risk of neurodegenerative dementia: a longitudinal follow-up study using a national sample cohort. Sci Rep.

[15] Kim S, Kim J, Kim K, Kim Y, Park Y, Baek S, Park SY, Yoon SY, Kwon HS, Cho YS (2013). Healthcare use and prescription patterns associated with adult asthma in Korea: analysis of the NHI claims database. Allergy.

[16] Choi HG, Kim JH, Park JY, Hwang YI, Jang SH, Jung KS (2019). Association between asthma and depression: a national cohort study. J Allergy Clin Immunol Pract.

[17] Choi HG, Lee JK, Kong IG, Lim H, Kim SY (2019). Osteoporosis increases the risk of benign paroxysmal positional vertigo: a nested case-control study using a national sample cohort. Eur Arch Otorhinolaryngol.

[18] Yu JS, Lee CJ, Lee HS, Kim J, Han Y, Ahn K, Lee SI (2012). Prevalence of atopic dermatitis in Korea: analysis by using national statistics. J Korean Med Sci.

[19] Kim SY, Park B, Lim H, Kim M, Kong IG, Choi HG (2019). Gastroesophageal reflux disease increases the risk of chronic rhinosinusitis: a nested case-control study using a national sample cohort. Int Forum Allergy Rhinol.

[20] Eldeirawi K, Persky VW (2004). History of ear infections and prevalence of asthma in a national sample of children aged 2 to 11 years: the Third National Health and Nutrition Examination Survey, 1988 to 1994. Chest.

[21] Cunsolo E, Marchioni D, Leo G, Incorvaia C, Presutti L (2010). Functional anatomy of the Eustachian tube. Int J Immunopathol Pharmacol.

[22] Seo Y, Nonaka M, Tagaya E, Tamaoki J, Yoshihara T (2015). Eosinophilic otitis media is associated with asthma severity and smoking history. J Oto-rhino-laryngol Relat Special.

[23] Toivonen L, Forsstrom V, Waris M, Peltola V (2019). Acute respiratory infections in early childhood and risk of asthma at age 7 years. J Allergy Clin Immunol.

[24] van Meel ER, den Dekker HT, Elbert NJ, Jansen PW, Moll HA, Reiss IK, de Jongste JC, Jaddoe VWV, Duijts L (2018). A population-based prospective cohort study examining the influence of early-life respiratory tract infections on school-age lung function and asthma. Thorax.

[25] Bonnelykke K, Vissing NH, Sevelsted A, Johnston SL, Bisgaard H (2015). Association between respiratory infections in early life and later asthma is independent of virus type. J Allergy Clin Immunol.

[26] Feldman AS, Hell Y, Moore ML, Hershenson MB, Hartert TV (2015). Toward primary prevention of asthma. Reviewing the evidence for early-life respiratory viral infections as modifiable risk factors to prevent childhood asthma. Am J Respirat Crit Care Med..

[27] Teo SM, Mok D, Pham K, Kusel M, Serralha M, Troy N, Holt BJ, Hales BJ, Walker ML, Hollams E (2015). The infant nasopharyngeal microbiome impacts severity of lower respiratory infection and risk of asthma development. Cell Host Microbe.

[28] Martines F, Martinciglio G, Martines E, Bentivegna D (2010). The role of atopy in otitis media with effusion among primary school children: audiological investigation. Eur Arch Oto-rhino-laryngol.

[29] Roditi RE, Veling M, Shin JJ (2016). Age: An effect modifier of the association between allergic rhinitis and Otitis media with effusion. Laryngoscope.

[30] Roditi RE, Shin JJ (2018). The influence of age on the relationship between allergic rhinitis and otitis media. Curr Allergy Asthma Rep.

[31] Duah V, Huang Z, Val S, DeMason C, Poley M, Preciado D (2016). Younger patients with COME are more likely to have mucoid middle ear fluid containing mucin MUC5B. Int J Pediatr Otorhinolaryngol.

[32] Rosenfeld RM, Shin JJ, Schwartz SR, Coggins R, Gagnon L, Hackell JM, Hoelting D, Hunter LL, Kummer AW, Payne SC (2016). Clinical Practice Guideline: Otitis Media with Effusion (Update). Otolaryngol Head Neck Surg.

