# Supplementary material for: Bidirectional association between asthma and otitis media in children
Source: Allergy Asthma Clin Immunol. 2021 Jan 9;17:7. doi: 10.1186/s13223-020-00500-7 (PMC7796599; doi:10.1186/s13223-020-00500-7)
Supplement: Supplementary file 1 — Additional file 1. File S1. Description: study population and data collection. [file 13223_2020_500_MOESM1_ESM.docx]

## **S1 description: Study Population and Data Collection**

The present study was based on data from the Korean National Health Insurance Service-National Sample Cohort (NHIS-NSC). To minimize nonsampling errors, the samples were extracted exactly from the mother population of the Korean NHIS. Approximately 2% (one million) of the whole Korean population (50 million) was chosen. These sampled data represent the mother population obtained by stratified systemic sampling methods. The selected population was classified at 1,476 levels (age [18 categories], sex [2 categories], and income level [41 categories]). The validity of the sample cohort was confirmed in a previous study [1]. The specific procedures for sampling the cohort are described on the website of the National Health Insurance Sharing Service [2]. This cohort database comprises detailed medical management histories for every participant from 2002 to 2013: (i) personal information, (ii) health insurance claim codes (procedures and prescriptions), (iii) diagnostic codes using the International Classification of Disease-10 (ICD-10), (iv) death records from the Korean National Statistical Office (using the Korean Standard Classification of Disease), (v) socio-economic data (residence and income), and (vi) medical examination data.

The precise population statistics were derived using these NHIS data. The Korean Health Insurance System is mandatory for all Koreans. Using the Health Insurance Review & Assessment (HIRA) system, all medical records can be traced without exception in Korea. Moreover, the possible overlapping of medical records was minimized, even when a patient moved to a different region of residence. All patients are registered using a 13-digit registration number in Korea. The 13-digit registration number is legally issued to every Korean for life. The death of a patient must be reported to an administrative entity before a funeral is conducted. The date and cause of death are listed on a death certificate issued by medical doctors.

Age groups were categorized in 5-year intervals: 20–24, 25–29, 30–34…, and 85+ years old. Fourteen age groups were classified. The income groups were initially classified into 41 classes (one health aid class, 20 self-employment health insurance classes, and 20 employment health insurance classes). These groups were then integrated into 5 classes (class 1 [lowest income]-5 [highest income]). Region of residence was classified into 16 areas in accordance with administrative areas. These regions were further classified into urban (Seoul, Busan, Daegu, Incheon, Gwangju, Daejeon, and Ulsan) and rural (Gyeonggi, Gangwon, Chungcheongbuk, Chungcheongnam, Jeollabuk, Jeollanam, Gyeongsangbuk, Gyeongsangnam, and Jeju) areas.

**Reference**

1. Lee J, Lee JS, Park SH, Shin SA, Kim K: **Cohort Profile: The National Health Insurance Service-National Sample Cohort (NHIS-NSC), South Korea**. *Int J Epidemiol* 2017, **46**(2):e15.

2. **National Health Insurance Sharing Service** [<http://nhiss.nhis.or.kr>]
