# Supplementary material for: Bidirectional association between asthma and otitis media in children
Source: Allergy Asthma Clin Immunol. 2021 Jan 9;17:7. doi: 10.1186/s13223-020-00500-7 (PMC7796599; doi:10.1186/s13223-020-00500-7)
Supplement: Supplementary file 2 — Additional file 2. Table S2. General characteristics of participants. [file 13223_2020_500_MOESM2_ESM.docx]

**Table S2** General Characteristics of Participants

| Characteristics | | | Study I | | | Study II | | | |
| --- | --- | --- | --- | --- | --- | --- | --- | --- | --- |
|  | |  | Asthma  (n, %) | Control I (n, %) | P-value* | | Otitis media (n, %) | Control II  (n, %) | P-value* |
| Age (years old) | | |  |  | 1.000 | |  |  | 1.000 |
|  | 5-9 | | 10,837 (28.1) | 10,837 (28.1) |  | | 18,024 (40.1) | 18,024 (40.1) |  |
|  | 10-14 | | 3,828 (9.9) | 3,828 (9.9) |  | | 9,019 (20.1) | 9,019 (20.1) |  |
| Sex | | |  |  | 1.000 | |  |  | 1.000 |
|  | Male | | 8,116 (55.3) | 8,116 (55.3) |  | | 14,291 (52.9) | 14,291 (52.9) |  |
|  | Female | | 6,549 (44.7) | 6,549 (44.7) |  | | 12,752 (47.2) | 12,752 (47.2) |  |
| Income | | |  |  | 1.000 | |  |  | 1.000 |
|  | 1 (lowest) | | 1,009 (6.9) | 1,009 (6.9) |  | | 1,900 (7.0) | 1,900 (7.0) |  |
|  | 2 | | 1,717 (11.7) | 1,717 (11.7) |  | | 2,998 (11.1) | 2,998 (11.1) |  |
|  | 3 | | 2,920 (19.9) | 2,920 (19.9) |  | | 5,288 (19.6) | 5,288 (19.6) |  |
|  | 4 | | 4,475 (30.5) | 4,475 (30.5) |  | | 8,094 (29.9) | 8,094 (29.9) |  |
|  | 5 (highest) | | 4,544 (31.0) | 4,544 (31.0) |  | | 8,763 (32.4) | 8,763 (32.4) |  |
| Region of residence | | |  |  | 1.000 | |  |  | 1.000 |
|  | Urban | | 6,692 (45.6) | 6,692 (45.6) |  | | 12,576 (46.5) | 12,576 (46.5) |  |
|  | Rural | | 7,973 (54.4) | 7,973 (54.4) |  | | 14,467 (53.5) | 14,467 (53.5) |  |

*Chi-square test.
